# Epithelioid Sarcoma of Upper Extremity: Diagnostic Dilemma With Therapeutic Challenges

**DOI:** 10.7759/cureus.14156

**Published:** 2021-03-28

**Authors:** Leon Alexander

**Affiliations:** 1 Plastic & Reconstructive Surgery, Sheikh Khalifa Medical City, Abu Dhabi, ARE

**Keywords:** epithelioid sarcoma, hand, recurrence, multimodal therapy, soft tissue sarcoma

## Abstract

Epithelioid sarcoma is a rare, slow-growing, malignant tumor with multivariate presentation and a high rate of recurrence following surgery. Diagnosis is often missed or delayed due to its infrequent nature and confusing clinical and pathological presentation. This is compounded by the fact that this tumor is aggressive with a propensity for metastases without being detected. The treatment of this deadly tumor is controversial with no clear-cut consensus. The author presents a case of epithelioid sarcoma in the finger of a young patient with subsequent recurrence after surgery and a review of current literature pertaining to this aggressive tumor. This report would like to stress the importance of a multimodal approach in combatting this tumor as prompt diagnosis and aggressive therapy can significantly reduce the poor outcomes associated with this disease.

## Introduction

Enzinger (1970) first described epithelioid sarcoma (ES) as a rare, slow-growing, malignant tumor of the distal extremities with a paucity of symptoms and a propensity for recurrence and metastasis. This variant of sarcoma accounts for less than 1% of all soft tissue sarcomas [1,2]. Due to its multivariate presentation, diagnostic difficulty, aggressive nature, and therapeutic uncertainty, it has been described as a “great masquerader” and “a wolf in sheep's clothing” [3,4].

This tumor rarely affects children, and the most common site of occurrence in the pediatric age group is the head and neck [4]. In this report, we report a case of ES in a young patient’s finger, which was misdiagnosed initially as a neurofibroma and subsequently recurred after surgery. The relevant literature review about its diagnosis and treatment is discussed with an added emphasis on the multimodal and multidisciplinary approach required to successfully treat this tumor.

## Case presentation

An 18-year-old boy presented with a history of progressively increasing growth on his right ring finger. He had undergone excisional biopsy of the lesion 1 ½ years ago, which was performed at another hospital. The biopsy reported the lesion to be a schwannoma. He had associated pain and numbness of the affected finger, which was progressively increasing over the past six months.

**Figure 1:**
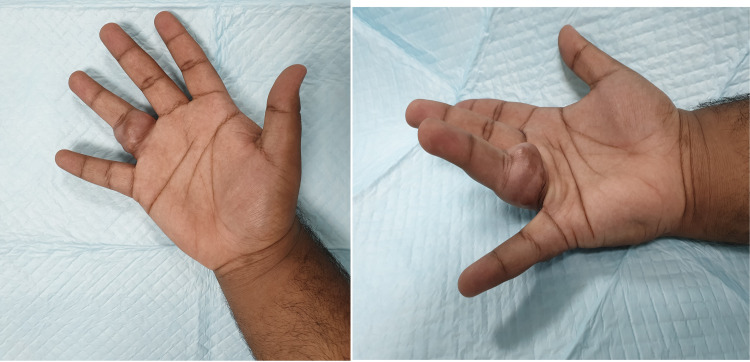
Pre-operative image of the hand showing an obvious tumor in the right ring finger.

On examination, there was a globular, lobulated swelling of size 6 x 3 cm encircling the proximal phalanx of the right ring finger extending over the mid axial lines on both sides (Figure 1).

It was tender on palpation and firm in consistency, and the skin over the swelling was not pinchable. The sensation was decreased over both radial and ulnar sides of the affected fingertip.

**Figure 2:**
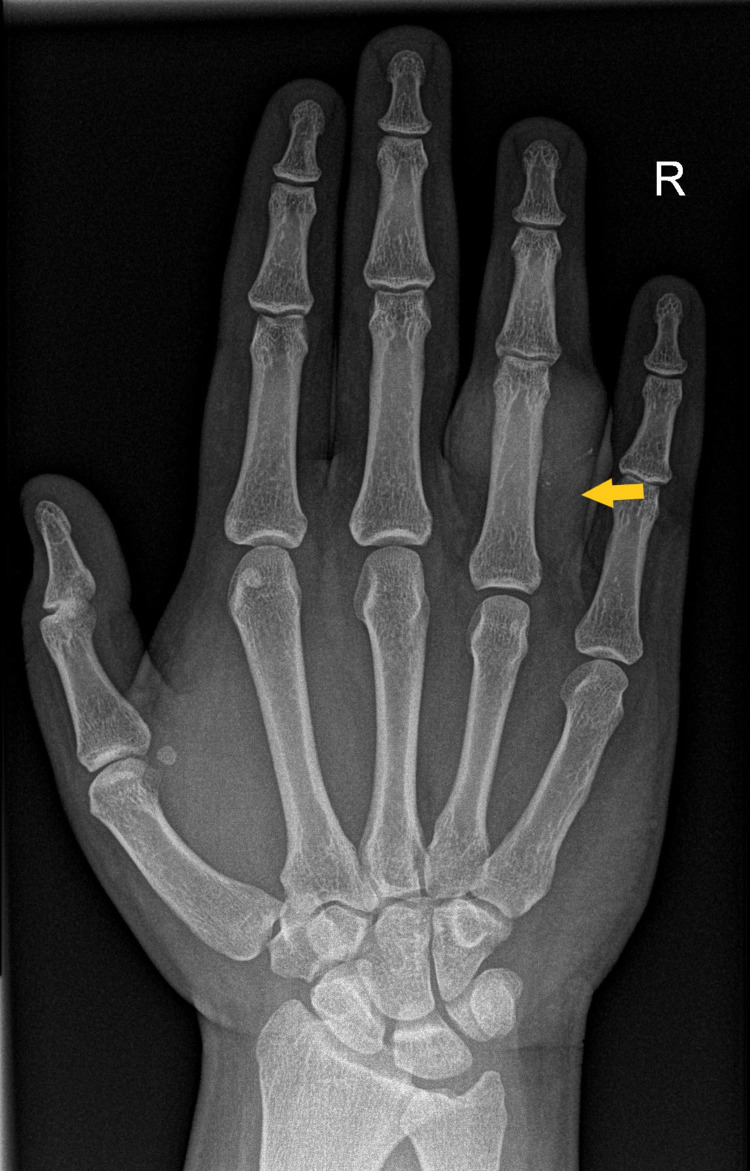
X-ray image showing soft tissue involvement of the affected ring finger (yellow arrow).

X-ray of the right ring finger showed only soft tissue swelling with no bony involvement (Figure 2).

**Figure 3:**
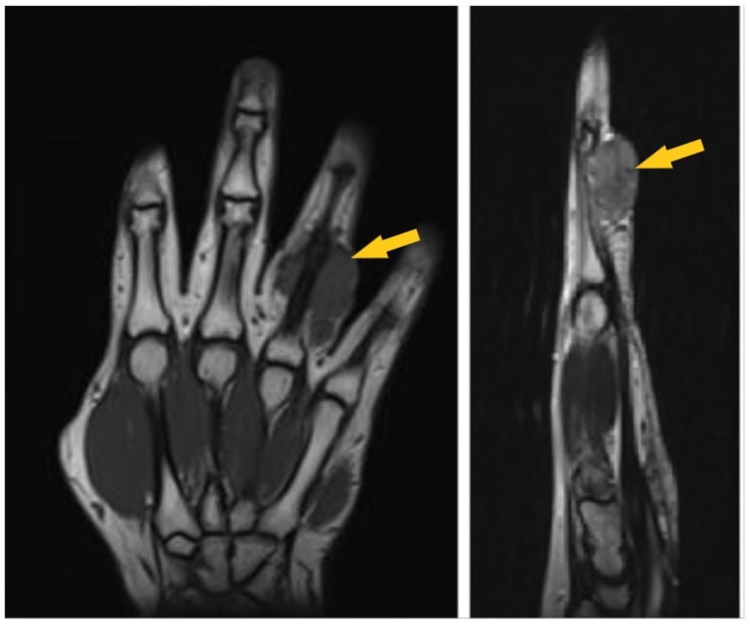
MRI (left: axial cut section; right: sagittal section) showing tumor in the right fourth finger with compression of the ulnar neurovascular bundle (yellow arrow).

MRI showed an irregular moderately enhancing soft tissue mass at the volar aspect of the right fourth (ring) finger at the level of the proximal phalanx, extending to both ulnar and radial surfaces, likely representing a benign soft tissue neoplasm or a peripheral nerve sheath tumor arising from the digital nerve on the ulnar side of the ring finger (Figure 3).

**Figure 4:**
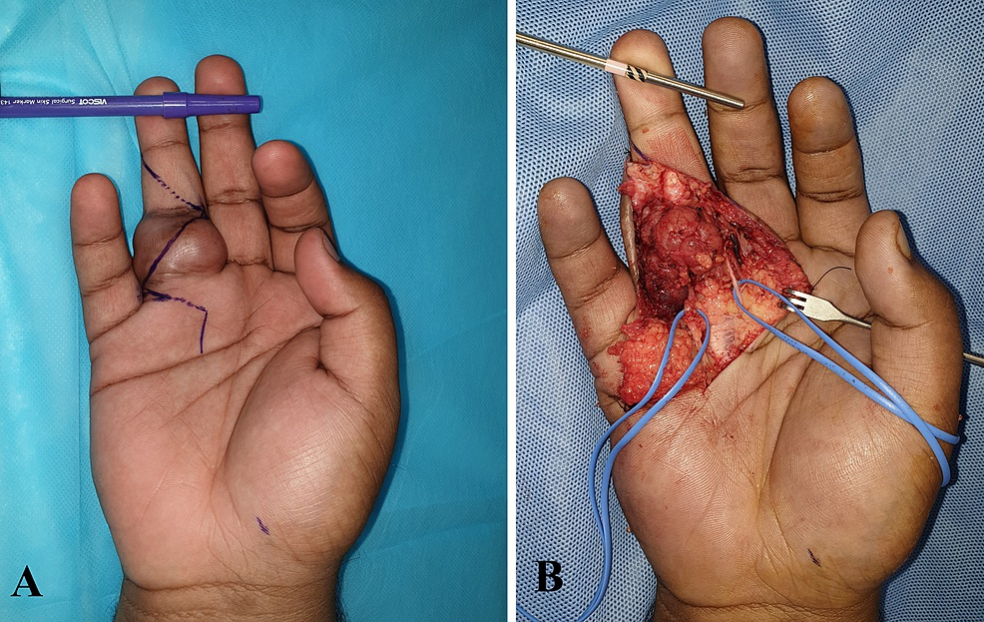
(A) Incision marking for volar Bruner flaps. (B) Dissection of the tumor and neurovascular bundles looped (blue vessel loops).

**Figure 5:**
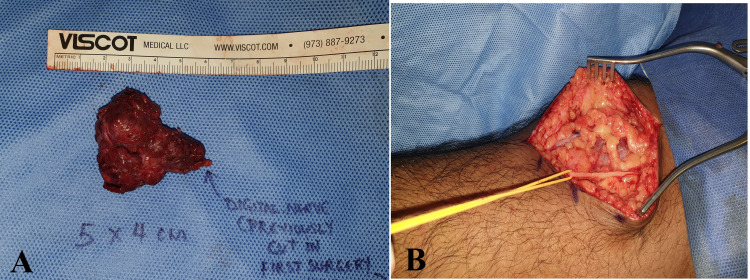
The tumor was excised completely. (B) Harvest of the nerve graft from the ipsilateral proximal forearm – lateral antebrachial cutaneous nerve (yellow loop).

**Figure 6:**
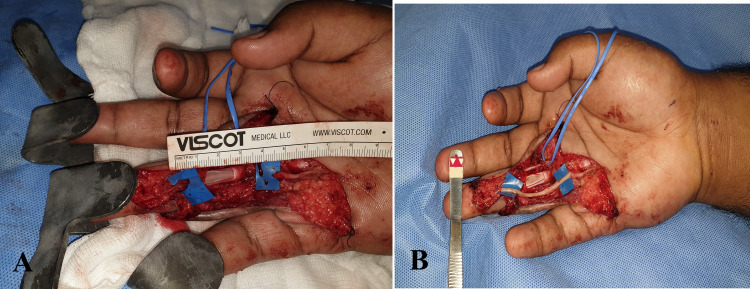
(A) Nerve gap after tumor excision in the ulnar branch of the digital nerve. (B) Nerve grafting was performed using the LABC nerve. LABC, lateral antebrachial cutaneous nerve

**Figure 7:**
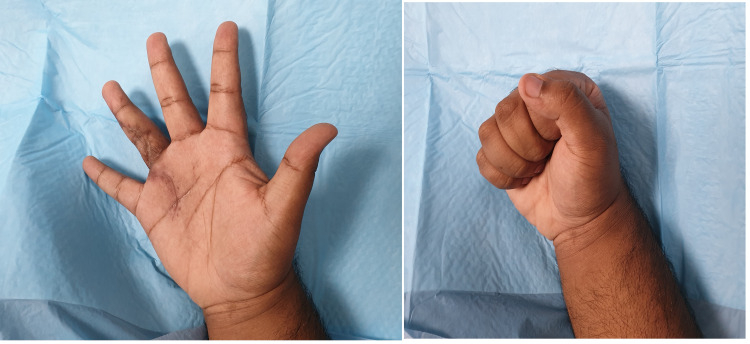
Post-operative result after three months.

The pre-operative diagnosis was a peripheral nerve sheath tumor arising from the digital nerve. The patient then underwent surgery. The intra-operative findings were nerve tumor of size 5 x 4 cm over the proximal and middle phalanx region of the right ring finger (Figure 4), the tumor was encasing the radial neurovascular pedicle, the ulnar branch of the digital nerve was found to be transected previously (in the first surgery), and the proximal end was found to be merged with the tumor and the distal end of the previously cut ulnar digital nerve was found just distal to dip joint with a stump neuroma (Figure 5). Hence, a nerve tumor excision, distal digital nerve stump neuroma excision, neurolysis, and nerve reconstruction with lateral antebrachial cutaneous nerve graft were performed (Figures 6, 7).

The post-operative biopsy reported it to be an ES and suggested immunohistochemical (IHC) studies to confirm this finding. IHC studies confirmed ES and it was found to be pan-CK (cytokeratin) positive, EMA (epithelial membrane antigen) positive, vimentin positive, and CD34 positive. The post-operative period was uneventful (Figure 7), but unfortunately, the patient failed to comply with the radiotherapy regimen as suggested by the oncologist. He subsequently developed a recurrence after six months and was advised ray amputation, which he refused and failed to show up for further follow-up.

## Discussion

ES is a rare, high-grade soft tissue sarcoma with a predilection for the distal aspect of extremities. However, on the hand, the incidence of ES is very rare, with the dorsum of the fingers being the most common site. It is the sixth most common soft tissue sarcoma of the upper extremity [5,6]. It is a slow-growing, indolent tumor and is frequently missed because of its initial benign presentation. It is commonly misdiagnosed as fibrosarcoma, synovial sarcoma, fibrous histiocytosarcoma, squamous cell carcinoma, Dupuytren’s disease, granulomatous processes, and rheumatoid nodules. It usually presents as a nodular lesion extending into the deep dermis, subcutaneous, and sometimes even the deeper subfascial structures. Once it extends into the deeper tissues, it encroaches along the tendon sheaths and neurovascular bundles causing compressive symptoms such as pain, paresthesia, restriction of movement, muscle weakness, and joint contractures [3,6-8].

ES can have a multivariate presentation in the upper extremity, but it can sometimes present with signs and symptoms of carpal tunnel syndrome due to compressive effects on the median nerve [6]. ES can also mimic Dupuytren's disease as it forms multiple painless nodules due to its growth along fascial and tendinous structures [3]. Similarly, it can cause extrinsic flexor tightness mimicking Volkmann ischemic contracture due to muscle infiltration. Any slow-growing, nodular fibrotic lesion of the upper extremity should be approached with caution, and a histological examination to rule out this malignant tumor must be conducted [9].

ES is rarely encountered in clinical practice, hence easily misdiagnosed initially as a benign condition, thereby delaying the definitive treatment. In our case, he was initially misdiagnosed as a case of neurofibroma. A simple excision was performed and after that the histology failed to diagnose an ES, thereby compounding the issue. Subsequently, he came to us with a recurrent tumor, and a repeat excisional biopsy and an immunohistochemical study confirmed the diagnosis of ES. Due to its diagnostic difficulty, an excisional biopsy and immunohistochemical studies must be performed to accurately diagnose this tumor.

The role of imaging in the diagnosis of ES is limited and radiography is of limited utility, although magnetic resonance imaging (MRI) could provide details of soft tissue infiltration and the tumor dimensions [9]. Once the diagnosis of ES has been established through biopsy, a staging CT scan of the thorax and abdomen should be performed followed by a positron emission tomography (PET) to scan for distant metastasis [1,9].

The treatment of ES of the extremity is challenging, especially in the hand due to its complex and intricate anatomy. The majority of ES are extra-compartmental and violate many tissue planes involving critical neurovascular structures. Optimal hand function and sound oncological principles with zero resection margins (R0 resection) must be adhered to when dealing with these tumors. ES is a surgically treatable disease; however, simple surgical excision of the tumor alone is inadequate due to a high risk of recurrence and metastasis [4,7,9].

The optimal treatment for ES involving the extremity entails wide en bloc excision or amputation with/without lymph node dissection followed by adjuvant chemotherapy and/or radiotherapy as it recurs and metastasizes even after wide excision. The prognosis of ES is dependent on recurrence, vascular invasion, and metastases (most commonly to the regional lymph nodes). Metastases have also been reported in the lungs, heart, pleura, liver, kidney, scalp, brain, pericardium, and bone [7,10,11].

The successful extirpation of ES from the upper extremity involves not only radical surgery to attain negative margins but also sophisticated, well-planned reconstruction using microsurgical principles (free flap reconstruction). Limb salvage is sometimes not possible in case of advanced or large tumors but must be attempted respecting the oncological principles of R0 resection. In cases where there is an involvement of neurovascular structures, complex reconstruction in the form of vein grafts and nerve grafts with concomitant flow-through free flaps as skin cover must be performed to salvage the limb and restore function. When radical resection entails the removal of the entire flexor muscle compartment of the forearm, a free functioning muscle transfer (gracilis free muscle flap) must be planned to restore flexion. Tendon transfers can also be performed if some flexors are preserved with intact extensor muscles to serve as donor tendons.

The addition of radical lymphadenectomy in the treatment of extremity sarcomas was studied by Fong et al., who concluded that lymphadenectomy increases the survival rates of patients with this tumor. Sentinel lymph node biopsy (SLNB) has been extensively studied in the management of melanoma and breast cancer, but its role in ES is uncertain. ES has a propensity for regional lymphatic spread and SLNB offers the advantage of a minimally invasive lymph-node staging procedure, which can improve the dismal prognosis and survival rates associated with this tumor. It also allows for the accurate early detection of micrometastasis, thereby sparing patients the morbidity associated with a regional lymphadenectomy [8,12,13].

What is the role of radiotherapy and chemotherapy in the treatment of this tumor? The roles of chemoradiotherapy in the treatment of ES is uncertain with no clear consensus [9,14]. The indications for adjuvant radiotherapy include incomplete tumor resection (R1 and R2 resection margins), recurrence, metastatic disease, and as a palliative measure in advanced tumors. Adjuvant chemotherapy (ifosfamide and doxorubicin) can be used in cases of advanced metastatic ES and can also be used as neoadjuvant therapy to reduce the tumor burden (shrink the tumor) before surgery. However, the role of chemotherapy in non-metastatic disease is unclear as the responses to chemotherapy are of short duration [12,15]. Future studies with long-term follow-up will be needed to establish the role of chemoradiotherapy in the treatment of ES.

Stang et al. reported a recurrence rate of up to 85% due to the growth of the tumor along the tendon sheath and concomitant neurovascular invasion. The recurrence rate for ES in the literature varies from 35% to 85% [8,11,16].

Amputation must be done for recurrent tumors; however, it does not seem to confer any benefit in patients with metastatic ES of the upper extremity. The overall five-year survival rates in the literature vary between 21% and 92%. Spillane et al. reported median 5- and 10- year survival rates of 70% and 42%. respectively. The median post metastatic survival rate was reported to be eight months [7,9,16].

## Conclusions

ESs are rare, malignant, indolent tumors with a preference for distal parts of the upper extremity. There is an initial diagnostic dilemma as they are confused with other benign conditions due to their slow-growing and multicentric nature. This often leads to underresection of these tumors with subsequent recurrence. There must be a high index of suspicion in diagnosing this tumor and a multidisciplinary, multimodal approach when treating this tumor. Surgery with wide local excision or amputation to attain negative margins is the mainstay in the treatment of this condition followed by adjuvant chemoradiotherapy, which is still experimental.
